# Improved Design of Anaerobic Digesters for Household Biogas Production in Indonesia: One Cow, One Digester, and One Hour of Cooking per Day

**DOI:** 10.1155/2014/318054

**Published:** 2014-03-13

**Authors:** Joseph G. Usack, Wiratni Wiratni, Largus T. Angenent

**Affiliations:** ^1^Biological and Environmental Engineering, Cornell University, Riley-Robb Hall, Ithaca, NY 14853, USA; ^2^Chemical Engineering, Gadjah Mada University, Jalan Grafika 2 Kampus UGM, Yogyakarta 55281, Indonesia

## Abstract

A government-sponsored initiative in Indonesia to design and implement low-cost anaerobic digestion systems resulted in 21 full-scale systems with the aim to satisfy the cooking fuel demands of rural households owning at least one cow. The full-scale design consisted of a 0.3 m diameter PVC pipe, which was operated as a conventional plug-flow system. The system generated enough methane to power a cooking stove for **∼**1 h. However, eventual clogging from solids accumulation inside the bioreactor proved to be a major drawback. Here, we improved the digester configuration to remedy clogging while maintaining system performance. Controlled experiments were performed using four 9-L laboratory-scale digesters operated at a temperature of 27 ± 1°C, a volatile solids loading rate of 2.0 g VS*·*L^−1^ *·*day^−1^, and a 21-day hydraulic retention time. Two of the digesters were replicates of the original design (control digesters), while the other two digesters included internal mixing or effluent recycle (experimental digesters). The performance of each digester was compared based on methane yields, VS removal efficiencies, and steady-state solids concentrations during an operating period of 311 days. Statistical analyses revealed that internal mixing and effluent recycling resulted in reduced solids accumulation compared to the controls without diminishing methane yields or solids removal efficiencies.

## 1. Introduction

The majority of Indonesia's people reside in remote, rural villages. Many of these villages lack access to conventional premium fuel sources such as electricity or fossil fuels. Consequently, many people are forced to use woody biomass as a source of heating energy and cooking fuel. Aside from adversely impacting local wooded ecosystems by deforestation, these practices pose serious human welfare issues since much of this biomass is burned indoors using poorly ventilated furnaces and cook stoves that generate high airborne particle matter (PM) concentrations. One way to overcome limited fuel availability and improve indoor air quality in rural areas is by generating biogas from animal manure using anaerobic digestion (AD) for use as a cooking fuel.

In an AD system, complex organic material is transformed and released as methane and carbon dioxide gas (i.e., biogas) by anaerobic bacteria and archaea in the absence of oxygen. Cow and other livestock manures have traditionally served as the feedstock material in rural AD systems; however, other organic wastes, such as crop residues or kitchen wastes, can be codigested with livestock manure to improve biogas yields [1, 2]. Biogas combustion does not produce PM emissions and for this reason is considered a clean-burning gas despite containing trace levels of toxic hydrogen sulfide (H_2_S). Indoor air quality assessments were conducted by Garfí et al. [3], and they found that H_2_S levels remained below detectable limits (<2 ppm) following five hours of biogas combustion [3]. They also found that PM emissions were reduced considerably when biogas was used in place of firewood [3]. Aside from improving air quality, the displacement of firewood with biogas has socioeconomic benefits because villagers (mainly women and children) spend less time collecting wood and more time pursuing other activities such as education and recreation.

In the past, the Indonesian government had supported the installation of community-scale digester systems in many remote villages in Indonesia. These digesters were based on the Chinese model and were constructed using concrete in a vertical fixed-dome configuration [4]. Unfortunately, the communities quickly abandoned many of these digesters due to improper construction, minimal institutional oversight, and poor training of the would-be operators. Indeed, these are just a few examples of the many barriers preventing the diffusion of biogas technology to rural communities. India and China are leading the application of biogas systems for rural areas with more than 40 million digesters installed [2]; however, these programs required over 50 years of sustained governmental sponsorship [4, 5].

Currently, the most popular type of small-scale digester is the Taiwanese model, which consists of a horizontal polyethylene tubular bag [6, 7]. This system has shown promising results in a study in Costa Rica for a mixture of swine waste and cooking oil [2] and in the Andes of Peru for dairy manure [8]. This type of digester is relatively inexpensive and easy to install, but direct sun exposure may reduce the flexibility of the polyethylene bag, making it more susceptible to leakage over time [2, 5]. Indeed, in a study comparing the fixed-dome digester (Chinese model) with the tubular bag digester (Taiwanese model), Garfí et al. [9] concluded that the main drawback of the fixed-dome digester was its relatively high capital cost and complex construction. While the tubular bag digester was less expensive and easier to install, it could incur considerably more maintenance cost through periodic replacement of the polyethylene bag [9].

**Figure 1:**
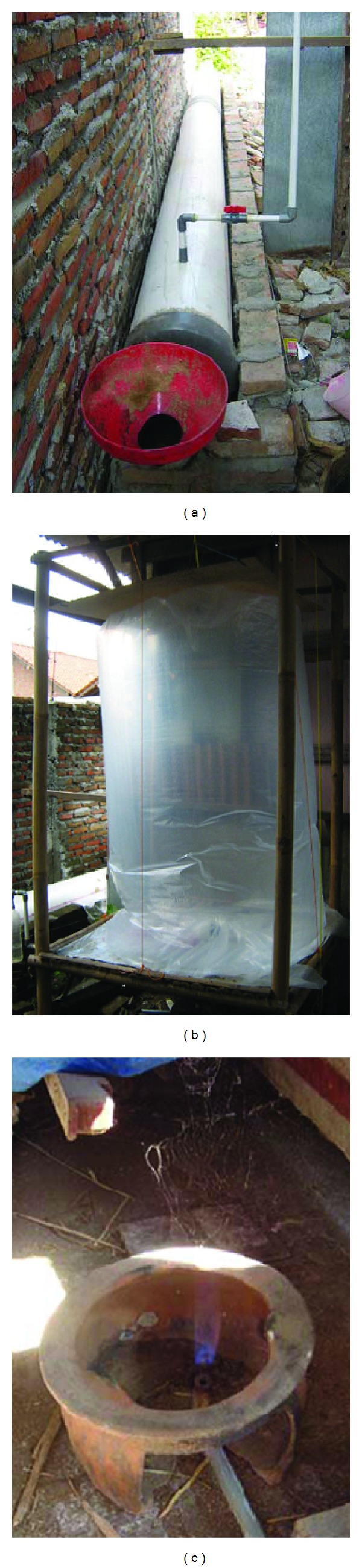
Field-scale Indonesian model digester. Pictures of a typical household biogas system: (a) the PVC digester, (b) the biogas collection bag, and (c) a traditional household cook stove fueled with biogas from the household digester.

Based on these historical lessons, the feasibility of a small-scale digester for use in individual households in Indonesia was explored as an intermediate step in developing a nationwide energy infrastructure based on AD. Therefore, a second wave of implementation was carried out under specific design constraints, which mandated that the digesters were: (i) inexpensive; (ii) easy to maintain; (iii) durable; and (iv) efficient in converting organic wastes into biogas. The design conceived by Purnomo and Pertiwiningrum [10] met these criteria and was selected. These digesters (Indonesian model) were constructed from 0.3 m diameter PVC tubes and operated as conventional plug-flow digesters (Figure 1). Ultimately, 21 of these digesters were installed and tested in rural areas of Indonesia with cow manure serving as feedstock (1–3 cows per household). These systems produced enough biogas on a daily basis to fuel a cook stove for ~1 hour. However, a major drawback of this design was a tendency to clog with solids internally over time, which caused diminished methane yields and interrupted operation. The repeated occurrence of clogging discouraged many villagers from its continued use. In fact, a follow-up survey of village participants revealed that 15 of the 21 digesters were abandoned within 3 years. In this study, our objective was to improve the Indonesian model digester design to reduce clogging while maintaining system performance.

Some degree of solids accumulation is inevitable in plug-flow and other flow-through systems, including the Taiwanese model, due to limited mixing within these systems. This can be especially problematic when cattle manure is used as the primary feedstock due to the high lignocellulosic biosolid content in this waste [11]. Indeed, these materials have a tendency to settle within the digester and are slow to degrade. As a result, the effective volume is reduced, and therefore the hydraulic retention time of the digester system is shortened, which leads to lower substrate conversion. Mechanical mixing can be used to resuspend settled solids and thereby mitigate solids accumulation and clogging; however, it also promotes hydraulic short circuiting within the plug-flow digester, which can reduce substrate conversion and consequently methane yield. In this study, we assessed the effectiveness of two manually operated, simple measures by introducing: (1) a mixing device and (2) effluent recycling as design modifications to reduce solids accumulation in the Indonesian model digester system.

## 2. Materials and Methods

### 2.1. Reactor Set-Up

**Figure 2:**
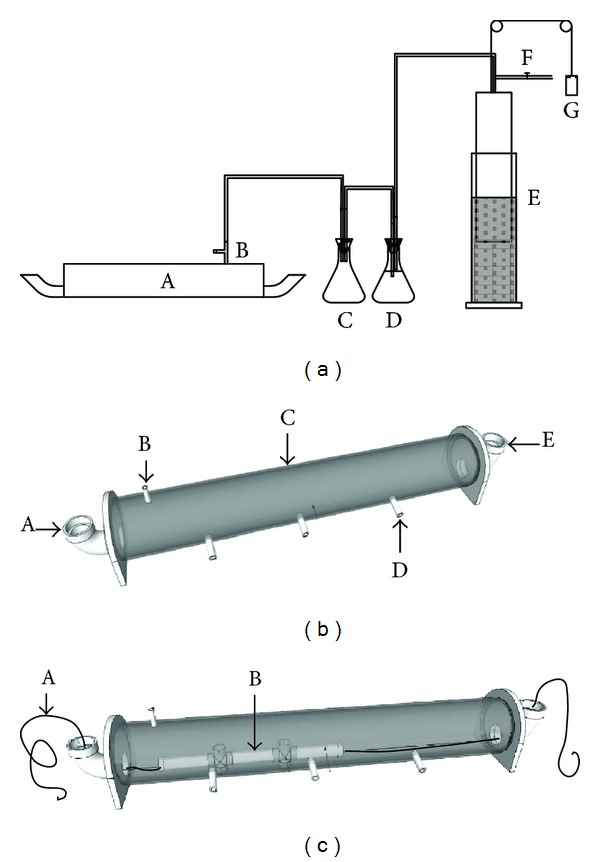
System diagram and digester designs: (a) system diagram showing the layout of the various components ((A) digester vessel; (B) gas sampling port; (C) foam trap; (D) bubbler; (E) displacement gas meter; (F) biogas exhaust; (G) gas meter counterweight)); (b) construction schematic of the laboratory-scale Indonesian model digester used for *R*_control-1_, *R*_control-2_, and *R*_recycle_ ((A) influent port - 3.8 cm I.D. PVC elbow; (B) biogas outlet line; (C) digester body - 10.0 cm I.D. PVC pipe; (D) sampling port; (E) effluent port - 3.8 cm I.D. PVC elbow)); (c) construction schematic for *R*_mixer_, which is the same as in (b), but a mixing rod has been added ((A) mixing wire and (B) internal mixing rod).

Four 9 L, laboratory-scale digesters were constructed from 1.3 m transparent PVC pipes with internal diameters of 10 cm. 3.8 cm PVC elbows served as the influent and effluent ports at both ends. Two digesters were scale replicates of the Indonesian model design and represented the controls (*R*_control-1_, *R*_control-2_) (Figures 2(a) and 2(b)). One experimental digester was equipped with an internal mixer (*R*_mixer_), consisting of a mixing rod that was operated manually by pulling a cord through the PVC elbows back and forth several times (Figure 2(c)). This digester was mixed immediately before and after feeding. The other experimental digester was operated with effluent recycle (*R*_recycle_) for which the first catchment of effluent was returned to the influent port following feeding. Otherwise, the reactor construction was the same as that for *R*_control-1_ and *R*_control-2_. Daily biogas production was measured using a tube-displacement gas meter (Figure 2(a)). The tube-displacement gas meters were calibrated using a standard gas meter (Actaris Meterfabriek, Delft, The Netherlands).

### 2.2. Reactor Operation

The inoculum consisted of dairy manure, which had been diluted to approximately 40 g VS·L^−1^. After achieving some methane production, all digesters were fed dairy manure that was diluted with tap water to an average volatile solids (VS) concentration of 42.1 ± 6.9 g VS·L^−1^. The manure was obtained from AA Dairy, Candor, NY. The fresh manure was screened (0.5 cm) before being stored at −20°C. The digesters were fed at an organic loading rate of ~2.0 g VS·L^−1^ *·*day^−1^ and a hydraulic residence time (HRT) of 21 days, each Monday, Wednesday, and Friday, for a total operating period of 311 days. The digesters were maintained at a temperature of 27 ± 1°C, established by the temperature controlled room in which they were placed. A one-way ANOVA test comparing mean solids concentrations (total solids [TS], volatile solids [VS], and fixed solids [FS]) fed to the digesters revealed no significant difference between systems (*P* value ≥ 0.2064, for all solid fractions), which substantiates comparisons between digester performances during steady-state conditions.

### 2.3. Analyses

Biogas production was measured daily, while pH and digester effluent solids concentrations were measured each feeding cycle. Additional measurements, such as total volatile fatty acid (TVFA) concentration, and soluble chemical oxygen demand were performed on a weekly basis. All analyses were conducted in accordance with APHA's standard methods [12]. Biogas methane content was measured using gas chromatography equipped with a flame ionization detector (FID) (SRI 8610 C, Torrance, CA, USA). Helium was used as carrier gas, with an inlet and detector temperature of 105°C, and a constant oven temperature of 40°C. Individual volatile fatty acids (iVFAs) concentrations were also measured by gas chromatography using FID (HP 5890 Series II). Helium was used as carrier gas, with an inlet and detector temperature of 200°C and 275°C, respectively. Individual VFA species were separated using a capillary column (NUKOL, Fused Silica Capillary Column, 15 m × 0.53 mm × 0.50 *μ*m film thickness; Supelco Inc., Bellefonte, PA), under a ramp temperature program (initial temperature 70°C for 2 minutes; temperature ramp 12°C per minute to 200°C; final temperature 200°C for 2 minutes). Due to the asymmetric feeding schedule, the data is shown as weekly averages to compensate for interweekly variation in performance metrics.

Statistical analysis of performance data was conducted using JMP PRO 10 Software (SAS Inc., USA). One-way analysis of variance was used to determine whether parametric variables differed between digester systems, while the Tukey-Kramer HSD model for comparing multiple means was used to make pairwise comparisons. The steady-state period used for statistical analysis was restricted to the period following day 63, representing three HRTs after inoculation. On a few occasions, the biogas outlet line became blocked with sludge and we excluded performance data corresponding to these incidents from statistical comparisons. We allowed for one complete HRT before assuming steady-state conditions following these events. Note that the plugging issues experienced in this study (at the biogas outlet line) were different in nature from the plugging issues experienced with the large-scale systems (internally) and were due to size scaling effects. Indeed, the headspace height in the laboratory-scale systems is 10 cm less than in the field-scale systems, which allowed foam within the digester to contact the biogas outlet port.

## 3. Results and Discussion

### 3.1. Digester Start-Up Dynamics

**Figure 3:**
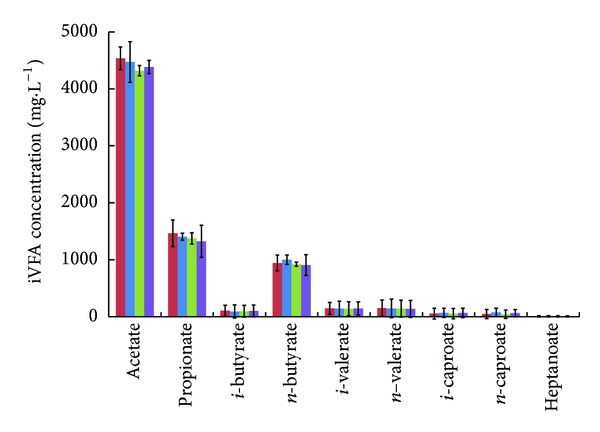
Postinoculation concentration of individual volatile fatty acids. Concentration of individual volatile fatty acid species after inoculation with dairy manure; *R*_control-1_ = red colored bar; *R*_control-2_ = blue colored bar; *R*_mixer_ = green colored bar; *R*_recycle_ = violet colored bar.

Each of the digesters exhibited minimal methane production for several weeks following inoculation with dilute dairy manure before feeding even started. To diagnose this problem, samples from each digester were taken and analyzed for individual VFA species. As expected, high concentrations of VFAs were detected in all systems (Figure 3). The dominant VFA molecule was acetate (C2) (4.32–4.54 g·L^−1^); however, longer-chain VFAs were also present at fairly high concentrations, which suggests possible inhibition to methanogens [13]. Of the longer-chain VFAs detected, propionate (C3) (1.32–1.46 g·L^−1^) and butyrate (C4) (0.91–1.00 g·L^−1^) were most prevalent. In conventional AD systems, roughly 75% of methane formation occurs through acetoclastic methanogenesis with the remainder following from hydrogenotrophic methanogenesis [14]. Acetate is generated by acetogenic bacteria through syntrophic oxidation and *β*-oxidation of longer-chain VFAs [15]. The acetate must be removed as quickly as it is produced by acetoclastic methanogenesis or syntrophic acetate oxidation to prevent product accumulation and allow *β*-oxidation metabolism to proceed.

**Figure 4:**
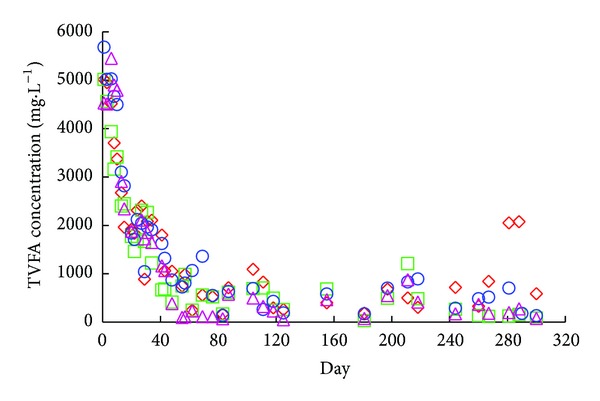
Total volatile fatty acid concentrations for all digester systems during the operation period; *R*_control-1_ = ⋄; *R*_control-2_ = ○; *R*_mixer_ = □; *R*_recycle_ = △.

The observed VFA accumulation after inoculation was most likely caused by organic overloading of the system by the manure, whereby the rate of acetate formation exceeded the utilization rate by methanogens. The methanogens may have been inhibited or, because of their relatively slow growth rate, were present at too low concentration. Eventually, methane production did initiate and the accumulated VFAs began to be consumed at which point, feeding and normal operation began (i.e., day 0). Within 63 days of the operating period (after feeding had commenced), the VFAs were reduced to concentrations characteristic of stable conditions (Figure 4), suggesting that the biomass had acclimated and grown to concentrations sufficient enough to handle the organic loading rate that we employed [16]. The digesters maintained their stability for the remainder of the study.

**Figure 5:**
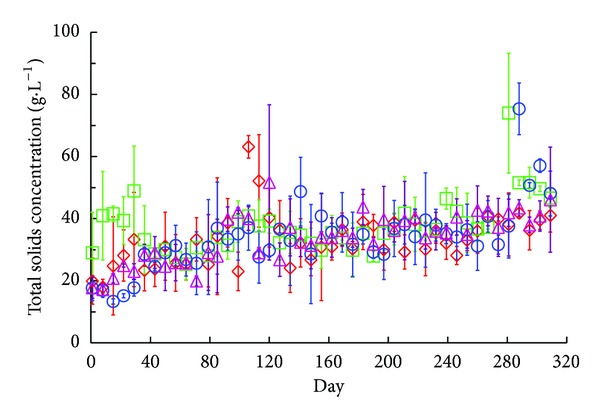
Total solids concentrations in the effluent of all digester systems during the operation period; *R*_control-1_ = ⋄; *R*_control-2_ = ○; *R*_mixer_ = □; *R*_recycle_ = △.

**Figure 6:**
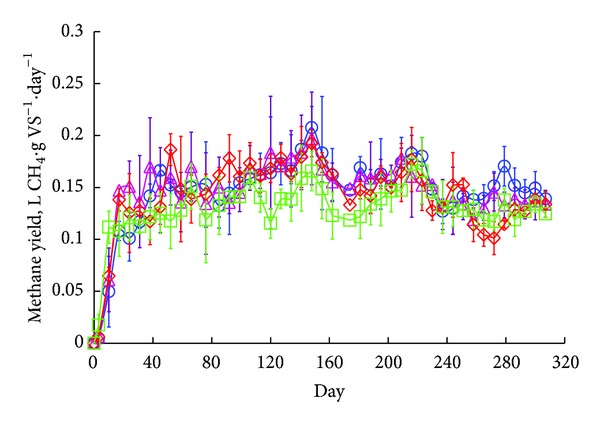
Average methane yield for all digester systems during the operation period; *R*_control-1_ = ⋄; *R*_control-2_ = ○; *R*_mixer_ = □; *R*_recycle_ = △.

During the start-up period (i.e., days 0–63), a noticeable difference in performance was observed between digester systems as each approached steady-state conditions. As anticipated, *R*_mixer_ produced greater effluent solids concentrations and lower methane yields initially compared to the other systems due to the effect of mixing (Figures 5 and 6). By mixing, *R*_mixer_ diverged from ideal plug-flow (PF) behavior in which discreet packets of fluid move serially from the inlet to the outlet with minimal fluid exchange (i.e., only molecular diffusion) to that of a completely mixed system in which reactor contents are uniformly dispersed [17]. Substrate conversion kinetics and performance are better in plug-flow systems compared to completely mixed systems because the biomass is subject to a higher initial substrate concentration and hydraulic short circuiting is minimized [17]. Despite these disadvantages, *R*_mixer_ eventually achieved methane yields and effluent solids concentrations comparable to the other digester systems. Finally, although effluent recycling would also encourage greater mixing, *R*_recycle_ did not show any lag in performance at the beginning of the operating period.

### 3.2. Comparison of Digester Performance during Steady-State Conditions

The average specific methane yields were comparable between digesters systems (Table 1), with the average steady-state values ranging from 0.14 to 0.15 L CH_4_·g VS^−1^·day^−1^. In fact, ANOVA revealed no difference in mean specific methane yield between digester systems (all pairwise comparisons yielded *P* values ≥ 0.1673) during the steady-state period (days 63–311). In comparison to other small-scale systems treating cattle manure, these specific methane yields are notably lower than those observed by Garfí et al. [18] (0.196 L CH_4_·g VS^−1^·day^−1^; tubular configuration) and by Ferrer et al. [8] (0.192–0.226 L CH_4_·g VS^−1^·day^−1^; tubular configuration). However, in these cases the organic loading rates were considerably lower (0.22–1.29 g VS·L^−1^·day^−1^) and hydraulic retention periods were considerable longer (60–90 days) than those employed in this study, which would contribute to greater specific methane yields. When comparing biogas yields, however, the digesters used in this study produced substantially more biogas (0.550 to 0.630 L_biogas_·L_reactor_^−1^·day^−1^) compared to Garfí et al. [18] and Ferrer et al. [8] (0.120 and 0.070–0.470 L_biogas_·L_reactor_^−1^·day^−1^, resp.).

Similarly, no difference in mean TS, VS, and FS removal efficiencies was found between digesters (all one-way ANOVAs, *P* values ≥ 0.07), with VS removal efficiencies ranging from 28.8 to 35.7%. This degree of VS reduction is typical for AD systems treating cattle manure in laboratory settings at mesophilic temperatures [1, 19] but is likely greater than experienced in the field, which may have lower average temperatures and suffer from diurnal and seasonal fluctuations. In this study, we observed consistently low VFA concentrations and fairly high VS concentrations in the effluent during steady-state operation, which suggest that the system was limited by hydrolysis [20]. This is typical for anaerobic digestion of cattle manure, since a large fraction of the organic material is cellulosic in composition (i.e., 56% of VS), rendering it less biodegradable [11].

### 3.3. Effect of Mixing and Effluent Recycling on Solids Retention

Despite similar TS, VS, and FS removal efficiencies between digesters, there were marked differences in retained solids concentrations. We define retained solids here as the quantity of solids accumulated within the digester during the operating period, which persists despite fluctuations in mass inputs and outputs. The retained solids concentrations (as TS) within the digesters were measured at the end of the study period and were equal to 71.4 ± 2.8, 74.3 ± 0.6, 81.1 ± 1.0, and 84.1 ± 1.0 g·L^−1^, for *R*_mixer_, *R*_recycle_, *R*_control-2_, and *R*_control-1_, respectively (Table 1). Analysis by ANOVA resulted in a strong rejection of the null hypothesis that the mean retained solids concentrations were equal between the experimental and control digesters (all pairwise *P* values ≤ 0.0006). Indeed, *R*_mixer_ and *R*_recycle_ significantly reduced the amount of retained solids in the digesters compared to the controls. Yet, the difference between mixing and recycling conditions was not statistically significant. This implies that both mixing and effluent recycling are effective techniques and either can be used to reduce solids retention within the digester.

The mechanism driving the reduction in retained solids was clearly observed in both *R*_mixer_ and *R*_recycle_. Indeed, the mixing rod in *R*_mixer_ acted to dislodge and temporarily resuspend the solids that had settled between feeding cycles, which allowed more to escape in the effluent, while, in *R*_recycle_, the very process of pouring in the recycled effluent created additional turbulence, which allowed greater expulsion of solids by bulk transport. Although there was never an instance of clogging in any of the digester systems during the study, we suspect that a reduction of retained solids would translate to fewer cases of clogging in the field. It is also possible that such mixing techniques would improve digester performance in cases where the solids retention time is excessive and HRT is shortened [21, 22]. Insight from this study also suggests that the inclination of the reactor system (i.e., the degree angle from horizontal) is an important design parameter, particularly in preventing clogging. Greater inclinations would create higher shear velocities within the digester when the influent is introduced, thereby disrupting bed formation along the digester length; however, it would also promote thicker beds near the digester outlets where clogging occurs. In our study, the digesters were oriented <5° from horizontal, which may explain the absence of clogging. Finally, although specific instances of clogging are not mentioned in the literature for studies involving Taiwanese model digesters, potential problems associated with solid retention, especially for shortened HRTs, are frequently cited in [2, 7, 9, 18, 23]. In Lansing et al. [2], VS accumulations of 46.7% above the influent VS concentration were observed, which is lower than those observed in this study (*R*_mixer_ = 53.9%, *R*_recycle_ = 55.3%, *R*_control-2_ = 69.6%, and *R*_control-1_ = 77.8%). In the Lansing et al. study, however, swine manure served as the substrate and the OLR was quite low in comparison (0.34 g VS·L^−1^·day^−1^), while the HRT was much longer (40 days). Mechanical mixing and effluent recycling were effective in reducing solids retention in the laboratory-scale digesters used in this study. We expect that similar reductions in solids retention could be achieved in the Taiwanese model digester when these mixing techniques are employed. Nevertheless, long-term field trials involving the Indonesian, Taiwanese, and Chinese models should be conducted to validate their effectiveness when used in the field by native operators.

## 4. Conclusion

Small-scale inexpensive anaerobic digester systems have become an essential technology for many rural households in developing countries, such as Indonesia, China, and India, providing a reliable source of cooking fuel. The Taiwanese and Indonesian model digester systems are simple flow-through systems, which are intrinsically susceptible to solids accumulation and clogging, which cause reductions in methane yield and require extra maintenance. The results from this study suggest that the use of digester mixing or effluent recycling is an effective way to mitigate solids accumulation in these systems without significantly jeopardizing their performance in terms of methane yield or solids removal efficiency making them more reliable and less of a burden to operate because of less maintenance. Our results show that the retained solids concentrations greatly exceed the influent solids concentration even for the experimental digesters. This implies that internal solids accumulation is inevitable in these digester systems. Therefore, these digesters may still need to be cleaned periodically; albeit we predict that the digesters with digester mixing or effluent recycling will need to be cleaned less often than the control digesters. Thus, the use of mixing or recycling would improve the system design and would likely reduce maintenance by village operators considerably. This may encourage greater adoption of this technology in developing countries, thereby improving social well-being in these communities. However, field tests at scale are still needed to determine whether the decrease in retained solids concentration is substantial enough to affect a meaningful decrease in clogging and yield more successful applications in rural areas.

## Figures and Tables

**Table 1:** Performance and chemical characteristics of the dairy manure feedstock and digester influents and effluents.

	Dairy manure	*R*_control-1_		*R*_control-2_		*R*_mixer_		*R*_recycle_	
		Influent	Effluent	Influent	Effluent	Influent	Effluent	Influent	Effluent
Total solids (TS)	134.3 ± 20.1	48.9 ± 7.5	33.8 ± 5.4	49.0 ± 6.7	34.5 ± 3.6	47.3 ± 6.9	36.2 ± 4.2	49.1 ± 6.9	36.9 ± 4.9
g·L^−1^	*n* = 7	*n* = 90	*n* = 25	*n* = 81	*n* = 24	*n* = 84	*n* = 26	*n* = 100	*n* = 29
Volatile solids (VS)	117.1 ± 19.1	42.4 ± 7.2	27.0 ± 5.3	42.5 ± 6.7	27.7 ± 3.5	40.8 ± 6.8	29.4 ± 4.1	42.5 ± 6.8	29.9 ± 4.7
g·L^−1^	*n* = 7	*n* = 90	*n* = 25	*n* = 81	*n* = 24	*n* = 84	*n* = 26	*n* = 100	*n* = 29
Fixed solids (FS)	17.2 ± 12.3	6.4 ± 0.9	6.8 ± 0.5	6.7 ± 0.8	6.9 ± 0.5	6.5 ± 0.8	6.8 ± 0.4	6.6 ± 1.0	6.5 ± 0.9
g·L^−1^	*n* = 7	*n* = 90	*n* = 25	*n* = 81	*n* = 24	*n* = 84	*n* = 26	*n* = 100	*n* = 29
Total COD	n.d.	60.9 ± 11.0	41.1 ± 11.9	56.6 ± 10.2	33.6 ± 9.5	64.1 ± 14.5	35.0 ± 10.5	56.6 ± 10.8	38.2 ± 14.9
g O_2_·L^−1^		*n* = 8	*n* = 9	*n* = 8	*n* = 9	*n* = 8	*n* = 9	*n* = 8	*n* = 9
Soluble COD	n.d.	9.3 ± 2.7	3.3 ± 1.4	8.6 ± 2.5	3.5 ± 1.4	9.1 ± 3.3	3.2 ± 1.5	9.1 ± 3.9	3.1 ± 1.3
g O_2_·L^−1^		*n* = 29	*n* = 20	*n* = 29	*n* = 20	*n* = 29	*n* = 20	*n* = 29	*n* = 20
Volatile fatty acids	2.00 ± 0.50	n.d.	0.67 ± 0.54	n.d.	0.51 ± 0.32	n.d.	0.41 ± 0.30	n.d.	0.30 ± 0.22
g CH_3_COOH·L^−1^	*n* = 3		*n* = 19		*n* = 19		*n* = 19		*n* = 19
pH	n.d.	7.65 ± 0.48	7.36 ± 0.20	7.57 ± 0.46	7.36 ± 0.20	7.59 ± 0.45	7.33 ± 0.21	7.54 ± 0.45	7.33 ± 0.20
		*n* = 112	*n* = 99	*n* = 114	*n* = 88	*n* = 112	*n* = 86	*n* = 114	*n* = 88
Retained solids	n.d.	84.1 ± 1.0		81.1 ± 1.0		71.4 ± 2.8		74.3 ± 0.6	
g TS·L_Reactor_^−1^		*n* = 3		*n* = 3		*n* = 3		*n* = 3	
Methane content	n.d.	58.0 ± 3.0		58.1 ± 2.1		59.0 ± 2.1		59.4 ± 2.1	
%		*n* = 32		*n* = 32		*n* = 32		*n* = 32	
Biogas production	n.d.	0.600 ± 0.070		0.630 ± 0.100		0.550 ± 0.090		0.590 ± 0.080	
L_Biogas_ · L_Reactor_^−1^·day^−1^		*n* = 96		*n* = 98		*n* = 98		*n* = 98	
Methane yield	n.d.	0.150 ± 0.030		0.150 ± 0.020		0.140 ± 0.020		0.150 ± 0.030	
L_CH_4__·g VS^−1^·day^−1^		*n* = 82		*n* = 76		*n* = 79		*n* = 94	

n.d.: not determined.
